# Testing effects of partner support and use of oral contraception during relationship formation on severity of nausea and vomiting in pregnancy

**DOI:** 10.1186/s12884-023-05468-x

**Published:** 2023-03-14

**Authors:** Kateřina Roberts, Jan Havlíček, Šárka Kaňková, Kateřina Klapilová, S. Craig Roberts

**Affiliations:** 1 Department of Zoology, Faculty of Science, Charles University, Prague, Czech Republic; 2 Department of Philosophy and History of Science, Faculty of Science, Charles University, Prague, Czech Republic; 3 Faculty of Humanities, Charles University, Prague, Czech Republic; 4 National Institute of Mental Health, Klecany, Czech Republic; 5 Department of Psychology, University of Stirling, Stirling, UK

**Keywords:** Pregnancy sickness, Morning sickness, Oral contraceptive, Partner support, NVP

## Abstract

**Background:**

A recent study focusing on dietary predictors of nausea and vomiting in pregnancy (NVP) found that women with higher levels of partner support, and those who had used oral contraception (OC) when they met the father, both tended to report less severe NVP compared with previous non-users or those with less supportive partners. We provide a further test of these factors, using a large sample of women from four countries who retrospectively scored their NVP experience during their first pregnancy.

**Methods:**

We recruited women who had at least one child to participate in a retrospective online survey. In total 2321 women completed our questionnaire including items on demographics, hormonal contraception, NVP, and partner support. We used general linear models and path analysis to analyse our data.

**Results:**

Women who had used OC when they met the father of their first child tended to report lower levels of NVP, but the effect size was small and did not survive adding the participant’s country to the model. There was no relationship between NVP and partner support in couples who were still together, but there was a significant effect among those couples that had since separated: women whose ex-partner had been relatively supportive reported less severe NVP. Additional analyses showed that women who were older during their first pregnancy reported less severe NVP, and there were also robust differences between countries.

**Conclusions:**

These results provide further evidence for multiple influences on women’s experience of NVP symptoms, including levels of perceived partner support.

**Supplementary Information:**

The online version contains supplementary material available at 10.1186/s12884-023-05468-x.

## Introduction

Nausea and vomiting in pregnancy (NVP) affects women all around the world [1–3]. According to a recent meta-analysis, almost 70% of women experience NVP to at least some extent [4]. Although most common during the first trimester, symptoms very often persist throughout pregnancy [5]. Despite its prevalence, causes and mechanisms of NVP remain poorly understood. Some consider it to be a simple by-product of intense hormonal changes in pregnancy [6], including progesterone and estradiol but especially human chorionic gonadotropin (hCG) [2], which is produced by the trophoblast and subsequently by the placenta, with levels rising rapidly through the first trimester. However, in view of associations with beneficial effects, including higher birth weight and a lower probability of miscarriages, birth defects, pre-term deliveries and perinatal deaths [7], other authors suggest it has an adaptive function, such as causing compensatory placental growth or by reducing ingestion of harmful foods [8–11].

Recently, it was reported that women who used oral contraceptives (OC) when they met their partner experience lower NVP than non-users [12]. Fiurašková et al. [12] hypothesised that this could be due to within-couple genetic similarity, because OC-users may select relatively HLA-similar partners [13–15]. Conception with HLA-similar men could influence a cascade of responses, including reduced maternal immune response to the foetus [16, 17], less extensive uterine vasculature remodelling during placentation, reduced placental growth [18] and hence placental hCG production [19], and finally lower NVP [2, 20]. Alternatively, the effect of previous OC use on NVP might be mediated via partner support, based on previous evidence that women who met their partner while using OC were more generally satisfied with their relationship [21, 22]. Such support is important when dealing with distressful health issues [23], and good communication and perceived partner support are both connected with lower NVP [12, 24].

It should be noted that although Fiurašková et al. [12], reported that women’s OC use when meeting their partner (and father of their child) was associated with reduced NVP severity, this relationship was found during exploratory analysis as part of a larger study on dietary predictors of NVP [12]. Consequently, its robustness needs to be established by further confirmatory studies. The main aim of this study was therefore to test whether women using OC during partner choice do go on to experience lower levels of NVP. Additionally, we explored the possible role of partner support in NVP symptom severity. Based on the findings of previous studies, we expected that enhanced partner support would be connected with a lower level of NVP. To test these predictions, we used a large sample of women from whom we could collect the necessary data. These women were from the Czech Republic, Slovakia, UK, USA and Canada, and were part of a broader study primarily focusing on patterns of OC use and relationship satisfaction [21].

## Methods

### Participants

We used an open survey, available to each visitor to our survey site. To this site, we recruited women who had at least one child and we asked them a series of questions about their first pregnancy and the biological father of their first child. We used a variety of recruitment methods to maximise the sample size, including personal contacts and advertisements on pregnancy and parenthood discussion websites, for which there was no financial reward for taking part. In addition, to further boost sample size, we recruited a proportion of the participants through a research panel administered by Qualtrics.com. These panel participants received a small amount ($7) as compensation for their time. The Qualtrics system prevented participants from potentially attempting to create duplicate entries for financial reward so that there were no duplicate responses from the same IP address. The questionnaires were completed online via the Qualtrics platform and were in the Czech language for Czech and Slovakian participants, and in English for participants from the UK, USA and Canada. The survey was constructed in English, translated into Czech by a bilingual speaker, and then translated back into English by a different bilingual speaker. Based on this back-translation validation step, a small number of unclear items were identified and fixed before the surveys were launched. Before recruitment began, we pre-tested the surveys in both English and Czech with a small sample (*n* = 5 women in each language) to ensure that questions were understandable and to estimate the time taken to complete the survey.

Consistent with our ethical permission from the University of Liverpool Psychology Ethics Committee, participants provided informed consent by a mouse-click on the “I consent to take part” button at the end of the information sheet which formed the landing page of the survey. The information sheet explained that participation was anonymous and that there was no way to trace any information back to individual participants. All data were stored and coded according to the unique personal identifier automatically generated by the survey software.

In total, 3678 women participated in the study. Of these, 874 did not answer the question about hormonal contraception and were excluded. We further excluded women who reported another type of hormonal contraception than combined oral contraception (*n* = 273), women with lower age at pregnancy than 18 (*n* = 69), women who reported having a multiple birth (*n* = 91) and women whose country of origin was represented in our data by only one or very few participants (*n* = 50). In total, 2321 women who reported both their OC usage when meeting their partner and their level of NVP when pregnant with their first child were included in the analysis. Only women using combined oral contraception were counted as OC users, while non-users were women who reported not using any form of hormonal contraception and/or using other non-hormonal contraceptive methods such as condoms or a diaphragm. From these, 945 were OC users and 1376 were non-users. Participants were from the USA (*n* = 1122), the Czech Republic/Slovakia (*n* = 955), the UK (*n* = 153) and Canada (*n* = 91). At the time of testing, 1634 women were still in a relationship with the father of their child and 685 women were already separated (2 did not report this). Their average age was 37.8 and the average age at which the pregnancy occurred was 26.8 (18–48). In total, 1190 women gave birth to a boy and 1105 to a girl (48 did not report this).

### Measures

We asked participants if they used any type of hormonal contraception at the time when they began the relationship with the biological father of their first child. We also asked specifically what type and what brand of hormonal contraception they used. Their experience of NVP when expecting their first child was retrospectively assessed by two items, which were formulated as recall items based heavily on the wording and items in the Rhodes Index of Nausea, Vomiting and Retching [25], and adapting each of its main two forms of response option. The first item was: “*During your pregnancy, did you experience feelings of nausea, retching or vomiting which you attributed to “morning sickness“ or “pregnancy sickness”?*” with possible answers “*No*”, “*Mild*”, “*Moderate*”, “*Great*”, “*Severe*”. The second question focused on a frequency of vomiting in a typical day: “*At its peak, how often did you experience vomiting/retching in a typical day?*” with possible answers “*Never*”, “*Once*”, “*2–5 times*”, and “*More than 5 times*”. We coded the answers to both questions (1–5 and 1–4, respectively). Reliability analysis indicated high internal reliability (Cronbach’s α = 0.846). On this basis we summed these items to create a composite score of retrospectively scored NVP severity.

To measure partner support, we used Brown’s [26] measure of partner support behaviour. The questionnaire consists of 11 items and it is introduced as follows: “*How satisfied are you with the following aspects of the quality of your relationship with your partner?*”. The individual items include following statements: “*He shares similar experiences as me*”, “*He helps keep up my morale*”, “*He helps me out when I am in a pinch*”, “*He shows interest in my daily activities and problems*”, “*He goes out of his way to do special or thoughtful things for me*”, “*He allows me to talk about things that are very personal or private*”, “*He lets me know I am appreciated for the things I do for him*”, “*He tolerates my ups and downs and unusual behaviours*”, “*He takes me seriously when I have concerns*”, “*He says things that make my situation clearer and easier to understand*”, and “*He lets me know that he will be around if I need assistance*”. Each item is rated on a scale from 1 (“*Very dissatisfied*”) to 6 (“*Very satisfied*”) and then summed. For women still in a relationship with the father of their first child we used standard versions of the questionnaire, but for women who were separated we modified the wording of items by adding “*Thinking back about my ex-partner*…” at the beginning of each item.

### Statistical analysis

We first analysed potential predictors of NVP separately. As NVP severity was not normally distributed (Kolmogorov-Smirnov test, *P* < 0.001), we used non-parametric Spearman rank correlations where appropriate, or Mann-Whitney tests to analyse the effect of OC and sex of the child on women’s NVP scores. Next, for a more comprehensive analysis that accounted for other potential predictors, we used a univariate general linear model (GLM) with NVP score as the dependent variable. We included OC use when meeting the father as a fixed factor and added to the model other predictors that were found to have significant effects in the exploratory analysis. Although F-tests are often considered robust to deviation from normality only in certain circumstances [27, 28], recent simulation studies demonstrate that it is robust to Type 1 error regardless of either the severity of deviation from a normal distribution, the sample size, or unequal distribution between groups [29, 30]. Nonetheless, as recommended by Field [31], we checked and confirmed that the conclusions of our analyses were unchanged when we used a robust test with 20% trimmed means (for this, we used the WALRUS package in Jamovi). Note that because this technique permits inclusion of factors but not covariates, we controlled for Age when pregnant by regressing NVP severity on Age when pregnant (*r* = − 0.137, *P* < 0.001) and computing the standardised residuals for use as the dependent variable (i.e. NVP severity relative to Age when pregnant).

Additionally, we used path analysis to test possible direct or indirect effects of OC and Age when pregnant. For this we used GLM Mediation Model with NVP as dependent variable, age when pregnant as a mediator and OC when met as a factor. To test the effect of partner support (Brown’s measure of partner support behaviour), we performed further GLMs separately for women who were still with their partner and those who had separated from their partner, because their satisfaction scores cannot be directly compared.

Our sample sizes exceeded 787, which was the minimum identified by power analysis (G*Power) to detect a small effect (*d* = 0.1) with 80% power. All statistical tests were performed using Jamovi version 1.6.23. All *P* values were two-sided and we defined statistical significance with an alpha of 0.05.

## Results

### Predictors of NVP

Initial exploratory analyses revealed several significant associations with NVP severity (Table 1). Women who used OC when they met their partner reported less severe NVP than women who did not use OC at that time, and women pregnant with a girl reported more severe NVP compared to those who were pregnant with a boy. There was a negative correlation between NVP severity and Age when pregnant. Finally, there was a positive correlation between NVP severity and the time elapsed since the pregnancy.

**Table 1:** Descriptives and exploratory tests

		Mean (SD)	n	rho	p
OC use when met	User	4.28 (2.09)	944		< 0.001
	Non-user	4.59 (2.14)	1375		
Sex of child	Boy	4.38 (2.11)	1188		0.040
	Girl	4.55 (2.13)	1105		
Age when pregnant			2311	−0.147	< 0.001
Time since pregnancy			2311	0.130	< 0.001

Differences in NVP severity during the pregnancy leading to a woman’s first child were compared in relation to her OC use when she met the father, and the sex of the child (using Mann-Whitney U tests). In addition, age while pregnant and the time from the pregnancy to completing the survey were tested using Spearman correlations

We then used GLM to estimate the independent effect of OC use during relationship formation on NVP severity, while controlling for other variables. We did not include Time since pregnancy in this analysis, both because it was negatively correlated with Age when pregnant (rho = − 0.379, *P* < 0.001) and because a path analysis showed that the direct effect of Age when pregnant was far more influential on NVP severity than either its indirect effect via Time since pregnancy or the direct effect of Time since pregnancy (Supplemental Table 1, Supplemental Fig. 1). In the GLM model, the independent effects of OC use, Sex of child and Age when pregnant all remained statistically significant, while there was no significant OC use x Sex of child interaction (Table 2). We checked that these results were unlikely to be caused by non-normal distribution of the dependent variable by conducting a robust analysis using 20% trimmed means; in this analysis, both the effects of OC use (*Q* = 8.12, *P* = 0.005) and Sex of child (*Q* = 3.96, *P* = 0.047) remained significant.

**Table 2:** Outcome of a GLM to test independent effects on reported levels of NVP severity

Effect	Mean Square	df	F	p	η^2^
**OC use when met**	**26.90**	**1, 2281**	**6.13**	**0.013**	**0.003**
**Sex of child**	**19.39**	**1, 2281**	**4.42**	**0.036**	**0.002**
**Age when pregnant**	**176.51**	**1, 2281**	**40.21**	**<.001**	**0.017**
OC use when met x Sex of child	0.02	1, 2281	0.01	0.943	0.000

OC use when met refers to a woman’s use or non-use of oral contraception at the time when she met her partner. Statistically significant results are marked in bold

**Fig. 1:**
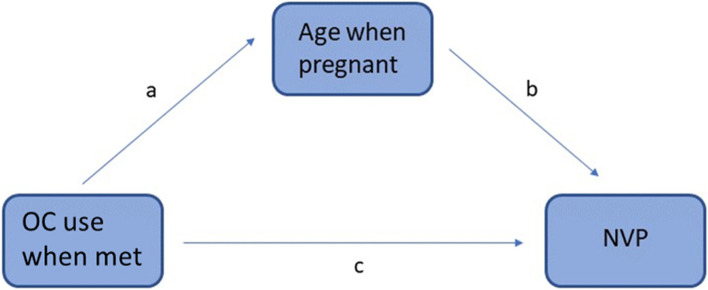
The scheme of mediation model. OC is the predictor variable, Age when pregnant is a mediator variable and NVP is the dependent variable

Although we found a relationship between women’s previous OC use and NVP, the effect of Age when pregnant was also significant and appeared stronger. However, we noticed that OC use when couples met also varied with Age when pregnant, with users being older on average (mean, SD: 27.6 years, 4.6, *n* = 939) than non-users (26.2 years, 5.4, *n* = 1374; *t* = 6.60, *P* < 0.001). For this reason, we also conducted a path analysis to investigate the relationships between these variables (Fig. 1). The results showed a significant direct effect of OC use, as well as an indirect effect of OC use through Age when pregnant, although the direct effect is much more pronounced (Table 3). This confirms that the effect of OC when couples met is not confounded by differences in age.

**Table 3:** Mediation estimates of the path analysis shown in Fig. 1

Effect	Label	Estimate	SE	Z	p	% Mediation
Indirect	a × b	− 0.0769	0.0169	− 4.55	<.001	25.4
Direct	c	−0.2258	0.0897	−2.52	0.012	74.6
Total	c + a × b	−0.3027	0.0896	−3.38	<.001	100.0

Finally, we tested for possible effects of the country from which participants came. Including Country in the model meant that the effect of previous OC use was no longer significant (*F*_1,2269_ = 1.77, *P* = 0.183); the only significant effects were now Age when pregnant (*F*_1,2269_ = 29.50, *P* < 0.001) and Country (*F*_3,2269_ = 31.51, *P* < 0.001). Indeed, comparison of NVP rates across countries showed that women from the Czech Republic/Slovakia (CZ/SK) reported significantly lower NVP severity (mean, SD; 3.90 ± 1.96) than women from either the UK (5.00 ± 2.27, *P* < 0.001) the USA (4.84 ± 2.12, post hoc Tukey test, *P* < 0.001) or Canada (4.78 ± 2.23, *P* < 0.001), while there were no significant differences between participants from the UK, USA and Canada. Based on these results, we divided the sample into two sub-samples (CZ/SK and UK/USA/Canada) and added this as a fixed factor (Country) to the original GLM. In this final analysis, which we believe to be the most robust test of our data, only the effect of Age when pregnant and Country remained significant (Table 4). We also ran the analyses separately for women from the two sub-samples. The results showed a robust and significant effect of Age when pregnant in both sub-samples, but the effects of OC and Sex of the child were no longer significant (Table 4).

**Table 4:** Effects on NVP severity in the whole sample (above) and the two geographical sub-samples (below)

	Mean Square	F	df	p	η^2^
***Whole sample***					
OC use when met	3.62	0.86	1, 2277	0.355	0.000
**Age when pregnant**	**109.71**	**26.00**	**1, 2277**	**< .001**	**0.011**
Sex of child	14.75	3.50	1, 2277	0.062	0.001
**Country**	**381.23**	**90.34**	**1, 2277**	**< .001**	**0.038**
OC use when met x Sex of child	0.14	0.03	1, 2277	0.854	0.000
OC use when met x Country	3.27	0.78	1, 2277	0.379	0.000
Sex of child x Country	1.02	0.24	1, 2277	0.623	0.000
OC use when met x Sex of child x Country	0.70	0.17	1, 2277	0.684	0.000
***Czech Republic / Slovakia***					
OC use when met	0.05	0.01	1, 920	0.904	0.000
**Age when pregnant**	**57.94**	**15.56**	**1, 920**	**<.001**	**0.017**
Sex of child	10.35	2.78	1, 920	0.096	0.003
OC use when met x Sex of child	0.05	0.01	1, 920	0.905	0.000
***UK / USA / Canada***					
OC use when met	8.74	1.92	1, 1356	0.166	0.001
**Age when pregnant**	**58.20**	**12.78**	**1, 1356**	**<.001**	**0.009**
Sex of child	4.64	1.02	1, 1356	0.313	0.001
OC use when met x Sex of child	0.84	0.18	1, 1356	0.668	0.000

Statistically significant results are marked in bold

### Partner support

Of the women included in the analyses above, 1634 were still in a relationship with the father and provided scores of current support, while 685 were no longer in that relationship and provided retrospective scores of partner support. For this reason, we analysed the association between NVP severity and levels of perceived partner support separately for these two groups. The same variables as in the previous models were included in this analysis, plus an additional variable – Partner support.

Among couples still together, there was a significant effect of Age when pregnant and Country on NVP severity, but we found no significant effect of Partner support, nor of either OC use during relationship formation or Sex of child (Table 5). Among separated couples, however, there was a significant effect of Partner support (*P* = 0.030), which was independent of the significant effect of Country. Women who reported relatively high levels of support from their ex-partner during pregnancy reported lower NVP severity. As before, the effects of OC use during relationship formation and Sex of child were not significant. Finally, in this sub-sample, the effect of Age when pregnant did not quite reach statistical significance (*P* = 0.082; Table 5).

**Table 5:** Effects on NVP severity in couples still together and couples who subsequently separated

	Mean Square	F	df	p	η^2^
***Couples still together***					
OC use when met	6.17	1.48	11,574	0.225	0.001
Sex of child	15.14	3.62	11,574	0.057	0.002
**Age when pregnant**	**86.98**	**20.80**	**11,574**	**< .001**	**0.012**
Partner support	0.11	0.026	11,574	0.872	0.000
**Country**	**277.97**	**66.47**	**11,574**	**< .001**	**0.040**
OC use when met x Sex of child	2.26	0.54	11,574	0.463	0.000
OC use when met x Country	1.20	0.29	11,574	0.592	0.000
Sex of child x Country	1.10	0.26	11,574	0.608	0.000
OC use when met x Sex of child x Country	1.90	0.45	11,574	0.501	0.000
***Couples who separated***					
OC use when met	0.73	0.17	1653	0.684	0.000
Sex of child	0.65	0.15	1653	0.700	0.000
Age when pregnant	13.40	3.04	1653	0.082	0.005
**Partner support**	**20.72**	**4.70**	**1653**	**0.030**	**0.007**
**Country**	**49.46**	**11.23**	**1653**	**< 0.001**	**0.017**
OC use when met x Sex of child	3.40	0.77	1653	0.380	0.001
OC use when met x Country	3.88	0.88	1653	0.349	0.001
Sex of child x Country	0.23	0.05	1653	0.818	0.000
OC use when met x Sex of child x Country	5.96	1.35	1653	0.245	0.002

Statistically significant results are marked in bold

## Discussion

In a large sample of women, we tested for possible associations between their OC use during relationship formation, or their satisfaction with the support they receive from their partner, and the perceived severity of NVP that they experienced in their first pregnancy. Based on previous findings, we expected that women who used OC during relationship formation and who expressed satisfaction with their partner’s support would report lower NVP severity. We found some initial support for the first prediction, but the effect was weak. The pattern was not present when we accounted for the women’s country of origin, nor when we tested it separately in samples of Czech/Slovakian women or women from the UK, the USA and Canada. Regarding the second prediction, we did find that women who reported higher satisfaction with their level of partner support had lower NVP, but this was only the case among women who had since separated from their partners; the effect was not present in those couples who remained together.

We found that there was a difference in levels of NVP between Czech/Slovakian women and those from the UK, the USA and Canada, with the former group having significantly lower NVP levels. We had not originally hypothesised a difference between these countries, but as the effect appeared to be strong, we included it in additional analyses. The discovery of this difference was important because it altered the conclusions we were able to draw about the apparent effect of oral contraceptive use at relationship formation. The initial analyses suggested that women experienced less severe NVP if they had met the father while using oral contraception, which would be consistent with our hypothesis that this may lead to relatively genetically similar partners, with potential corollary effects on NVP severity. The effect size was small, however, and this may be because it appears to be confounded by sample differences, such that Czech/Slovakian women who reported relatively less severe NVP were also more likely to have met the father while using OC (Supplemental Table 2), compared to women from the other countries. Our subsequent analyses in which we either include Country as a fixed factor in the model or analyse the two geographical sub-samples separately, confirm this. Our results thus expose and highlight the need for great caution in interpreting cross-national samples. If we had not included the effects of country, we would have reached a very different conclusion.

What lies behind this difference in perceived NVP severity is unclear but we can make some speculations. One suggestion is dietary differences between countries. For example, consumption of sugar-sweetened beverages is higher in the UK, the USA and Canada compared to the Czech Republic [32], and their consumption is associated with higher NVP levels [33]. Additionally, BMI could play a role, as higher pre-pregnancy BMI is associated with NVP levels [33, 34] and average BMI in the Czech Republic and Slovakia is lower than in the English-speaking countries in our sample, especially compared to the USA which represents the majority of this sub-sample [35]. An alternative form of explanation is a reporting difference, such that women from the Czech Republic and Slovakia experience an equivalent degree of NVP but tend to report it as less severe than those from the UK, the USA and Canada.

Although the apparent effect of previous OC use on subsequent NVP appears to be explained by other factors, this is not to say that it must be entirely non-existent. First, the confounds we discussed above are unlikely to be responsible for the positive association between NVP severity and OC use at relationhip formation reported by Fiurašková et al. [12], as their sample was predominantly from the UK or North America. Second, the literature shows clearly that NVP is affected by a wide variety of factors and, if OC use during partner choice does have some effect, this would likely be relatively small and could be easily overshadowed by other factors. Furthermore, we should bear in mind that OC use during partner choice, if it is a factor, is only (at best) a crude proxy for the hypothesised underlying mechanism, which is the level of HLA similarity between partners. There remains a need for further investigation of NVP levels in HLA-genotyped couples.

Our second focus of interest was whether NVP levels might be inversely associated with perceived levels of partner support [12], whether this is due to a causative influence of partner support on NVP level or to women with higher support tending to score their NVP as less severe. To test the effect of partner support, we analysed responses separately for women who were still in a relationship with their partner and for those who were no longer with their partner. Women who were still together with their partner reported their current level of partner’s support while those who were separated reported their received support retrospectively. We did not find a significant effect of partner support on NVP levels in women who were still together with their partners, but there was a protective effect on NVP of recalled partner support among those who had separated.

The fact that we did not find any effect of partner support in the group of women who were still with their partner could be caused by a ceiling effect, with relatively high scores of partner support across the sample (*still together* – mean, SD: 49.4, 11.9; *apart* – 30.1, 13.5), as well as lower variability in these scores compared to the sub-sample of women who had separated from their father (Levene’s test, *P* < 0.05). In the latter group, perhaps there was more variability in support even at the time of the pregnancy, which may have influenced women’s experience of NVP. Altogether then, our results indicated that partner support may play a role, at least to some extent, in either affecting the level of NVP or in the subjective perception of NVP symptoms.

We also found several other factors to be correlated with NVP severity scores. First, women’s age when pregnant was consistently a significant predictor of NVP. Women who were pregnant at a younger age had higher NVP severity scores than women who were pregnant at an older age. This inverse relationship between age and NVP is consistent across many studies [36–38]. Second, sex of the child was significantly associated with NVP scores in the initial analysis, although the effect size was small and disappeared in analyses that separately examined women still, or no longer, in a relationship with the father. This pattern matches previous evidence which is also somewhat inconsistent: several studies suggest that bearing a female foetus is associated with higher NVP ([39–42], but other studies do not find a sex difference [43].

### Criticisms

Although our sample was relatively large and targeted specifically at women’s first pregnancy, our approach introduced certain limitations which must be acknowledged. First, women provided retrospective scores of their NVP symptoms and severity. Retrospective reports are prone to memory-related biases [44] and an alternative approach would be to sample currently pregnant women, as we did previously [12]. There is, however, evidence that women can accurately and reliably report distant (10 to 15 years) events in their pregnancy [45] (although NVP was not among the tested variables in that study). We are aware that because of the possibility of memory-related bias, we need to interpret our results carefully.

Second, this retrospective approach meant that we could not use a standard measure of NVP severity such as the Rhodes Index [25], because this asks women about their symptoms over the preceding 12 hours. Instead, we asked women to provide scores on two items that dealt with their recalled experience over the entire pregnancy. (It would be interesting, in a future study, to examine the correlation between Rhodes Index scores measured in the first trimester with scores on our retrospective items measured sometime after the pregnancy, but this was beyond the scope of this study.) Although these two criticisms compel us to be cautious about our results, the finding that age of pregnancy consistently predicted NVP scores, as it does in many previous studies, provides some reassurance about the accuracy of recall. Furthermore, an advantage of looking back over the whole pregnancy, compared to Rhodes Index responses, is that we circumvent the problem of varying onset and persistence of NVP symptoms: although NVP is most common in the first trimester, some women experience symptoms later or even throughout their pregnancy [12].

Another criticism is that possible recall bias may also have impacted on women’s ratings of partner support. Approximately 30% of the sample were no longer in a relationship with the father of the first child. This meant that we had to perform the analysis of partner support separately for women who were still with, or no longer with, their partners. It also meant that women rated partner support slightly differently: those who were still together provided a rating of current support, while those who were separated assessed support retrospectively. We were therefore careful to analyse these sub-samples separately. An advantage of the approach, however, was that it revealed an interesting difference between the subsamples in the effect of partner support, such that an effect of reduced partner support may be easier to detect in couples whose relationship was destined soon to end.

## Conclusion

Although NVP is a widely occurring phenomenon in pregnancy, its causes and mechanisms are still not clear. Our study was motivated by two new potential predictors revealed in a previous exploratory analysis [12], but we did not find strong supporting evidence for these in this sample. We did not find strong evidence for an effect of OC use during relationship formation once the confounding effect of country of origin was taken into account. We did find some support for an effect of poor partner support on NVP levels, but only in couples who had separated when the survey was competed. In any case, it is likely that such effects would be rather small, considering how complex a phenomenon NVP is. More investigation is needed, including in samples of women who are currently pregnant and with more sensitive questionnaires, while also considering possible underlying mechanisms such as hormone levels and HLA similarity of partners.

## Supplementary Information

**Additional file 1: Supplemental Table 1.** Results of mediation model. **Supplemental Fig. 1.** The scheme of the mediation model. **Supplemental Table 2.** Descriptives of two sub-samples of women according to country.

## Data Availability

De-identified data from this study are available upon reasonable request from the corresponding author, or from the University of Stirling DataSTORRE at the following webpage: http://hdl.handle.net/11667/203. No specific materials were used in this study beyond published scales or items described in the methods section of this paper.

## Abbreviations

OC: Oral contraception/oral contraceptive
NVP: Nausea and vomiting in pregnancy
hCG: Human chorionic gonadotropin
GLM: General linear model
BMI: Body mass index
HLA: Human leukocyte antigen

## References

[1] Kramer J, Bowen A, Stewart N, Muhajarine N (2013). Nausea and vomiting of pregnancy: prevalence, severity and relation to psychosocial health. Amer J Matern Child Nurs.

[2] Lee NM, Saha S (2011). Nausea and vomiting of pregnancy. Gastroenterol Clin N Am.

[3] Pepper G, Roberts SC (2006). Rates of nausea and vomiting in pregnancy and dietary characteristics across populations. Proc R Soc B Biol Sci.

[4] Einarson TR, Piwko C, Koren G (2013). Quantifying the global rates of nausea and vomiting of pregnancy: a meta analysis. J Popul Ther Clin Pharmacol.

[5] Lindseth G, Vari P (2005). Nausea and vomiting in late pregnancy. Health Care Women Int.

[6] Lagiou P, Tamimi R, Mucci LA, Trichopoulos D, Adami HO, Hsieh CC (2003). Nausea and vomiting in pregnancy in relation to prolactin, estrogens, and progesterone: a prospective study. Obstet Gynecol.

[7] Patil CL, Abrams ET, Steinmetz AR, Young SL (2012). Appetite sensations and nausea and vomiting in pregnancy: an overview of the explanations. Ecol Food Nutr.

[8] Huxley RR (2000). Nausea and vomiting in early pregnancy: its role in placental development. Obstet Gynecol.

[9] Lumey LH (1998). Compensatory placental growth after restricted maternal nutrition in early pregnancy. Placenta..

[10] Hook EB (1978). Dietary cravings aversions during. Am J Clin Nutr.

[11] Profet M. Pregnancy sickness as adaptation: a deterrent to maternal ingestion of teratogens. In: Barkow JH, Cosmides L, Tooby J, editors. The Adapted Mind: Evolutionary Psychology and the generation of culture. Oxford: Oxford University Press; 1992. p. 327–65.

[12] Fiurašková K, Havlíček J, Roberts SC (2021). Dietary and psychosocial correlates of nausea and vomiting in pregnancy. Food Qual Prefer.

[13] Havlíček J, Roberts SC (2009). MHC-correlated mate choice in humans: a review. Psychoneuroendocrinology..

[14] Wedekind C, Seebeck T, Bettens F, Paepke AJ (1995). MHC-dependent mate preferences in humans. Proc R Soc B Biol Sci.

[15] Roberts SC, Gosling LM, Carter V, Petrie M (2008). MHC-correlated odour preferences in humans and the use of oral contraceptives. Proc R Soc B Biol Sci.

[16] Havlíček J, Winternitz J, Roberts SC (2020). Major histocompatibility complex-associated odour preferences and human mate choice: near and far horizons. Philos Trans R Soc B: Biol Sci.

[17] Moffett A, Loke C (2006). Immunology of placentation in eutherian mammals. Nat Rev Immunol.

[18] Madeja Z, Yadi H, Apps R, Boulenouar S, Roper SJ, Gardner L (2011). Paternal MHC expression on mouse trophoblast affects uterine vascularization and fetal growth. Proc Natl Acad Sci U S A.

[19] Korevaar TIM, Steegers EAP, de Rijke YB, Schalekamp-Timmermans S, Visser WE, Hofman A (2015). Reference ranges and determinants of total hCG levels during pregnancy: the generation R study. Eur J Epidemiol.

[20] Forbes S (2002). Pregnancy sickness and embryo quality. Trends Ecol Evol.

[21] Roberts SC, Klapilová K, Little AC, Burriss RP, Jones BC, DeBruine LM (2012). Relationship satisfaction and outcome in women who meet their partner while using oral contraception. Proc R Soc B Biol Sci.

[22] Roberts SC, Little AC, Burriss RP, Cobey KD, Klapilová K, Havlíček J (2014). Partner choice, relationship satisfaction, and oral contraception: the congruency hypothesis. Psychol Sci.

[23] Revenson TA (1994). Social support and marital coping with chronic illness. Ann Behav Med.

[24] Iatrakis GM, Sakellaropoulos GG, Kourkoubas AH, Kabounia SE (1988). Vomiting and nausea in the first 12 weeks of pregnancy. Psychother Psychosom.

[25] Rhodes VA, McDaniel RW (1999). The index of nausea, vomiting, and retching: a new format of the index of nausea and vomiting. Oncol Nurs Forum.

[26] Brown MA (1986). Social support during pregnancy: a unidimensional or multidimensional construct?. Nurs Res.

[27] Montgomery DC (2017). Design and analysis of experiments.

[28] Winer BJ, Brown DR, Michels KM (1991). Statistical principles in experimental design.

[29] Schmider E, Ziegler M, Danay E, Beyer L, Bühner M (2010). Is it really robust? Reinvestigating the robustness of ANOVA against violations of the normal distribution assumption. Methodol Eur J Res Methods Behav Soc Sci.

[30] Blanca Mena MJ, Alarcón Postigo R, Arnau Gras J, Bono Cabré R, Bendayan R (2017). Non-normal data: Is ANOVA still a valid option?. Psicothema.

[31] Field A (2018). Discovering statistics using IBM SPSS statistics.

[32] Singh GM, Micha R, Khatibzadeh S, Shi P, Lim S, Andrews KG (2015). Global, regional, and national consumption of sugar-sweetened beverages, fruit juices, and milk: a systematic assessment of beverage intake in 187 countries. PLoS One.

[33] Chortatos A, Haugen M, Iversen PO, Vikanes Å, Magnus P, Veierød MB (2013). Nausea and vomiting in pregnancy: associations with maternal gestational diet and lifestyle factors in the Norwegian mother and child cohort study. BJOG..

[34] Klebanoff MA, Koslowe PA, Kaslow R, Rhoads GG (1985). Epidemiology of vomiting in early pregnancy. Obstet Gynecol.

[35] Abarca-Gómez L, Abdeen ZA, Hamid ZA, Abu-Rmeileh NM, Acosta-Cazares B, Acuin C (2017). Worldwide trends in body-mass index, underweight, overweight, and obesity from 1975 to 2016: a pooled analysis of 2416 population-based measurement studies in 128.9 million children, adolescents, and adults. Lancet.

[36] Källen B, Lundberg G, Åberg A (2003). Relationship between vitamin use, smoking, and nausea and vomiting of pregnancy. Acta Obstet Gynecol Scand.

[37] Dekkers GWF, Broeren MAC, Truijens SEM, Kop WJ, Pop VJM (2019). Hormonal and psychological factors in nausea and vomiting during pregnancy. Psychol Med.

[38] Fejzo MS, Trovik J, Grooten IJ, Sridharan K, Roseboom TJ, Vikanes Å (2019). Nausea and vomiting of pregnancy. Nat Rev Dis Primers.

[39] Danzer H, Braustein GD, Rasor J, Forsythe A, Wade ME (1980). Maternal serum human chorionic gonadotropin concentrations and fetal sex prediction. Fertil Steril.

[40] Czeizel AE, Puhó E (2004). Association between severe nausea and vomiting in pregnancy and lower rate of preterm births. Cent Eur J Public Health.

[41] Mitsuda N, Eitoku M, Maeda N, Fujieda M, Suganuma N (2019). Severity of nausea and vomiting in singleton and twin pregnancies in relation to fetal sex: the Japan environment and Children’s study (JECS). J Epidemiol.

[42] Young NR, La Rosa M, Mehr SA, Krasnow MM (2021). Does greater morning sickness predict carrying a girl? Analysis of nausea and vomiting during pregnancy from retrospective report. Arch Gynecol Obstet.

[43] Louik C, Hernandez-Diaz S, Werler MM, Mitchell AA (2006). Nausea and vomiting in pregnancy: maternal characteristics and risk factors. Paediatr Perinat Epidemiol.

[44] Coughlin SS (1990). Recall bias in epidemiologic studies. J Clin Epidemiol.

[45] Yawn BP, Suman VJ, Jacobsen SJ (1998). Maternal recall of distant pregnancy events. J Clin Epidemiol.

