# Supplementary material for: Testing effects of partner support and use of oral contraception during relationship formation on severity of nausea and vomiting in pregnancy
Source: BMC Pregnancy Childbirth. 2023 Mar 14;23:175. doi: 10.1186/s12884-023-05468-x (PMC10012454; doi:10.1186/s12884-023-05468-x)
Supplement: Supplementary file 1 — Additional file 1: Supplemental Table 1. Results of mediation model. Supplemental Fig. 1. The scheme of the mediation model. Supplemental Table 2. Descriptives of two sub-samples of women according to country. [file 12884_2023_5468_MOESM1_ESM.docx]

**SUPPLEMENTARY MATERIAL**

**Title: Testing effects of partner support and use of oral contraception during relationship formation on severity of nausea and vomiting in pregnancy**

**Authors: Kateřina Roberts^1^, Jan Havlíček^1^, Šárka Kaňková^2^, Kateřina Klapilová^3,4^, and S. Craig Roberts^5^**

^1^Dept of Zoology, Faculty of Science, Charles University, Prague, Czech Republic

^2^Dept of Philosophy and History of Science, Faculty of Science, Charles University, Prague, Czech Republic

^3^Faculty of Humanities, Charles University, Prague, Czech Republic

^4^National Institute of Mental Health, Klecany, Czech Republic

^5^Dept of Psychology, University of Stirling, UK

**Supplemental Table 1**

Results of mediation model

| Effect | Z | p | % Mediation |
| --- | --- | --- | --- |
| Indirect | -3.15 | 0.002 | 19.7 |
| Direct | -4.96 | <.001 | 80.3 |
| Total | -6.66 | <.001 | 100.0 |

| Path Estimates | Label | Estimate | SE | Z | p |
| --- | --- | --- | --- | --- | --- |
| age when pregnant → time since pregnancy | a | -7.82 | 0.40 | -19.80 | <.001 |
| time since pregnancy → NVP | b | 0.001 | 0.000 | 3.19 | 0.001 |
| age when pregnant → NVP | c | -0.05 | 0.009 | -4.96 | <.001 |

**Supplemental Figure 1**

The scheme of the mediation model**
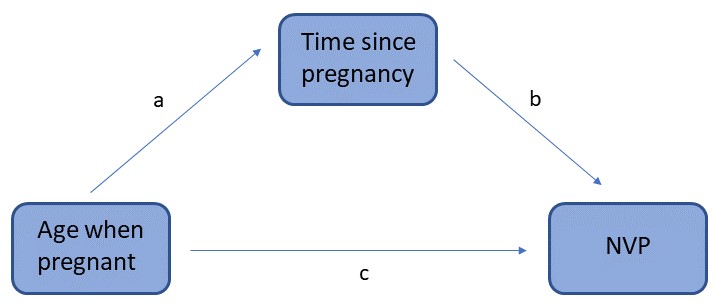
**

*Note.* Age when pregnant is predictor variable, time since pregnancy is mediator variable and NVP is dependent variable.

**Supplemental Table 2**

Descriptives of two sub-samples of women according to country

|  |  | Czech/Slovakia | UK/US/Canada | t | p |
| --- | --- | --- | --- | --- | --- |
| OC use when met | Yes  No | 486 (49.8 %)  489 (50.2 %) | 930 (66.1 %)  463 (33.9 %) | - | <.001 |
| Sex of child | Boy  Girl | 502 (52.9 %)  447 (47.1 %) | 696 (51 %)  668 (49 %) | - | 0.376 |
| Age when pregnant (yrs) | Mean (SD) | 27.8 (4.18) | 26.1 (5.67) | 7.72 | <.001 |
| Time since pregnant (yrs) | Mean (SD) | 7.6 (8.5) | 13.5 (8.3) | -16.6 | <.001 |
| Relationship with father | Still together  Separated | 807 (83 %)  165 (17 %) | 845 (61.8 %)  522 (38.2 %) | - | <.001 |
